# Patterns of Genome-Wide Diversity and Population Structure in the *Drosophila athabasca* Species Complex

**DOI:** 10.1093/molbev/msx134

**Published:** 2017-04-14

**Authors:** Karen M. Wong Miller, Ryan R. Bracewell, Michael B. Eisen, Doris Bachtrog

**Affiliations:** 1 Department of Integrative Biology, University of California Berkeley, Berkeley, CA; 2 Department of Molecular and Cell Biology, University of California Berkeley, Berkeley, CA; 3 Howard Hughes Medical Institute, University of California Berkeley, Berkeley, CA

**Keywords:** speciation, prezygotic isolation, population structure, *Drosophila*

## Abstract

The *Drosophila athabasca* species complex contains three recently diverged, prezygotically isolated semispecies (Western-Northern, Eastern-A, and Eastern-B) that are distributed across North America and share zones of sympatry. Inferences based on a handful of loci suggest that this complex might be an ideal system for studying the genetics of incipient speciation and the evolution of prezygotic isolating mechanisms, but patterns of differentiation have not been characterized systematically. Here, we assembled a draft genome for *D. athabasca* and analyze whole-genome re-sequencing data for 28 individuals from across the species range to characterize genome-wide patterns of diversity and population differentiation among semispecies. Patterns of differentiation on the X-chromosome vs. autosomes vary, with the X-chromosome showing better phylogenetic resolution and increased levels of between semispecies divergence. Despite low levels of overall differentiation and a lack of phylogenetic resolution of the autosomes for the most closely related semispecies, individuals do exhibit distinct genetic clustering. Demographic analyses provide some support for a model of isolation with migration within *D. athabasca*, with divergence times <20 kya. The young divergence times of the semispecies of *D. athabasca*, together with strong levels of sexual isolation, makes them a promising system for studying the evolution of prezygotic isolation and speciation.

## Introduction

Understanding the evolutionary forces and genetic patterns underlying the process of speciation is a major aim in the field of evolutionary genetics. Studies utilizing *Drosophila* have greatly increased our understanding of speciation (Coyne and Orr 2004), especially the mechanisms contributing to postzygotic reproductive incompatibility (Presgraves 2010). However, we still know surprisingly little about the genetic forces that act during the initial stages of speciation. While the investigation of hybrid incompatibility factors is critical to understanding the evolution of reproductive isolation, such factors may not have been important early on during species divergence and may have only evolved secondarily (Orr 1995; Noor and Feder 2006; Sobel et al. 2010). By studying recently diverged populations, we increase the chances that the differences that we detect are actually directly responsible for reproductive isolation. Thus, investigating patterns of genomic divergence in incipient species is essential to uncover the evolutionary processes driving the emergence of reproductive isolation and new species.

**Fig. 1:**
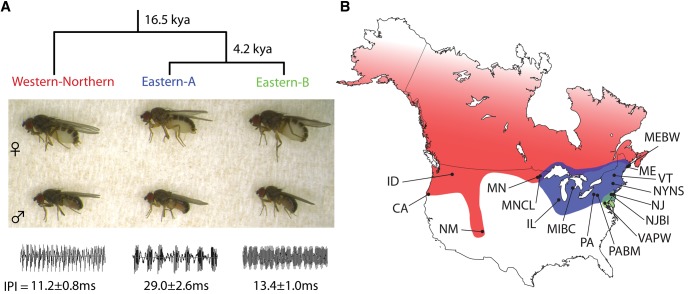
Overview of the *D. athabasca* semispecies complex and collection locations. (**a**) Semispecies are morphologically identical, but exhibit semispecies-specific courtship songs most easily quantified by differences in interpulse interval (IPI; the time from the end of a pulse to the start of the next) at High-Repetition-Rate (HRR) bursts. The average IPI along with standard deviations for each semispecies is indicated underneath a typical waveform. Western-Northern and Eastern-B exhibit similar IPIs, however their ranges do not overlap in nature **(b)**. Semispecies ranges are depicted by different colors, Western-Northern (WN) = red, Eastern-A (EA) = blue, Eastern-B (EB) = green. Abbreviations indicate sampling locations (see supplementary table S1, Supplementary Material online for details).

*Drosophila athabasca* is a North American species complex within the *obscura* group and *affinis* subgroup of *Drosophila*. The *affinis* subgroup consists of a young species radiation, with its oldest member, *D. azteca*, originating only 6 million years ago and with an average age of species in this subgroup of only 3.5 million years (Beckenbach et al. 1993). The *D. athabasca* complex is composed of three morphologically indistinguishable semispecies with partially overlapping ranges—Western-Northern, Eastern-A, and Eastern-B—that are thought to have diverged less than 25,000 years ago (Ford and Aquadro 1996) (fig. 1).

Despite their recent divergence, *D. athabasca* semispecies have already evolved strong prezygotic isolating barriers. In particular, laboratory crosses between *D. athabasca* semispecies produce fully viable and fertile offspring but have revealed a high degree of sexual isolation (Miller 1958; Miller and Westphal 1967; Miller et al. 1975; Yoon 1991; Ford et al. 1994; Yoon and Aquadro 1994; Ford and Aquadro 1996). During courtship, *Drosophila* males of many species produce species-specific courtship songs by vibrating their wings. Differences in courtship song, especially in the interpulse interval (IPI; the time from the end of a pulse to the start of the next) have been shown to be important for premating isolation in several *Drosophila* species (Saarikettu et al. 2005), and likely contribute to strong behavioral prezygotic isolation within the *D. athabasca* semispecies (Miller 1958; Miller et al. 1975; Yukilevich et al. 2016). *D. athabasca* has two types of song bursts: Low-Repetition-Rate (LRR) burst and High-Repetition-Rate (HRR) burst (Miller et al. 1975; Yoon 1991), and previous studies have revealed differences in courtship song, and in particular the IPI of HRR bursts, among semispecies (Yukilevich et al. 2016) (fig. 1*a*). Playback experiments of semispecies-specific songs increase the mating success of muted heterospecific *D. athabasca* males (Ford 1995; Yukilevich et al. 2016), demonstrating its importance in sexual isolation among semispecies.

Their geographic range and high degree of sexual isolation differentiate *D. athabasca* populations sufficiently for them to be designated as semispecies (Miller et al. 1975; Yukilevich et al. 2016). Thus, *D. athabasca* is a promising system for investigating the genetic mechanisms underlying a rapidly evolving prezygotic isolating barrier. However, despite the potential of *D. athabasca*, the species complex has not been widely studied at the DNA sequence level. Early studies in *D. athabasca* have examined allozyme and mtDNA differences between the semispecies, both of which concluded very recent genetic divergence between the semispecies, despite strong behavioral differences (Johnson 1978, 1985; Yoon and Aquadro 1994). A restriction site survey of variation at a few nuclear loci found greater differentiation between the three semispecies at X-linked genes than at autosomal genes (Ford and Aquadro 1996). However, beyond a handful of genes (Yoon 1991; Ford et al. 1994; Yoon and Aquadro 1994; Ford and Aquadro 1996), little is known about genome-wide patterns of molecular variation and differentiation within the species.

Here we utilize whole genome sequencing to study patterns of genomic differentiation in the *Drosophila athabasca* species complex. We assemble a draft genome and conduct a whole-genome population analysis of *D. athabasca* using polymorphism data from 28 individuals sampled from across the species range (fig. 1*b*), to describe patterns of genome-wide diversity and population structure and differentiation within *D. athabasca*. In particular, we examine whether our genomic data support the behavioral and geographic stratification of individuals into three semispecies, and compare patterns of nucleotide diversity within and divergence between semispecies on the X chromosome versus autosomes. Historical demography leaves characteristic signatures in the genome, and we use our population genomic data to conduct an analysis of current and ancestral population sizes, levels of gene flow, and the timing of population splits in the *D. athabasca* species complex. Finally, we discuss the potential of *D. athabasca* as a powerful model system for studying the early stages of speciation.

## Methods

### Collection of *Drosophila athabasca*

Flies were collected over banana bait during the summers of 2009–2011. To avoid creating artificial population structure as a result of sampling artifacts, we collected flies at 19 different locations widely spread across the *D. athabasca* species range (fig. 1*b*;supplementary table S1, Supplementary Material online). Over 800 iso-female lines were established from these collection sites, and we used Sanger sequencing of a mitochondrial DNA fragment to confirm which lines belonged to the *D. athabasca* species complex (*Cytochrome Oxidase II* gene; Fwd primer: GTTTAAGAGACCAGTACTTG; Rev primer: ATGGCAGATTAGTGCAATGG). A total of 404 *D. athabasca* lines were established in the lab (see supplementary table S1, Supplementary Material online for collection locations and number of lines).

### Courtship Song Assays

Courtship songs were recorded for 28 *D. athabasca* lines. Flies were reared at 20 **°**C on a 12 h light/12 h dark light cycle. Both male and female virgins were collected shortly following eclosion and aged in individual vials for 7–10 days under the same temperature and lighting conditions as during rearing. Recordings were captured by placing a single virgin male and virgin female in an Insectavox insect recording chamber (Gorczyca and Hall 1987). The Insectavox was connected to a RadioShack Mini Amplifier Speaker (Cat. No. 277-1008C) and MacBook Pro, and songs were recorded using the RAVEN software (Program 2011). All recordings were carried out at 21±1 **°**C. Three separate mating pairs were recorded for each line, and interpulse interval (IPI) from High-Repetition-Rate (HRR) song bursts was calculated directly from song waveforms as an average of the three pairs.

### Genome Assembly and Annotation

To create a reference genome assembly for *D. athabasca*, we extracted genomic DNA from a single strain (iso-female strain ID-10, Western-Northern) using the Puregene DNA Extraction Kit (Qiagen). We prepared a total of four genomic libraries using standard Illumina protocols, two short insert paired-end libraries with mean insert sizes of 91 bp (24 sd) and 340 bp (63 sd) from a genomic DNA extraction of 10 pooled females, and two additional mate-pair libraries with mean insert sizes of 2,046 bp (285 sd) and 4,813 bp (650 sd) from a genomic DNA extraction of 20 pooled females. The genomic libraries were sequenced for 101 bp from both ends, each on a lane of an Illumina Genome Analyzer II (GAII), resulting in a total of 54.0 million paired reads. The two long-insert mate-pair libraries were cropped to 36 bp to reduce the chances of reading over library construction breakpoints, as suggested by the manufacturer. Reads were screened and cropped for adapter and bacterial contamination, leaving a total of 53.0 million paired reads amounting to 4.7 Gb of sequence used in the assembly, or approximately 30× coverage of the genome. We assembled the reads using SOAPdenovo (Li et al. 2010) with a kmer size of 31, using mate-pair libraries for scaffolding. The GapCloser program within SOAPdenovo was used to close gaps. To assign scaffolds to Muller elements, scaffolds were BLASTed [(Altschul et al. 1990); −e 10e−20] to the *D. pseudoobscura* genome (version 2.25), throwing out any scaffolds without a hit.

To aid in genome annotation, we made three mRNAseq libraries using the *D. athabasca* reference strain, one with a pool of ten 5–10 days old female flies, another with a pool of ten 5–10 days old male flies, and a final with a pool of 10 mixed sex third-instar larvae. We extracted mRNA using the TRIzol extraction method (Life Technologies) followed by poly-A selection using Dynabeads (Life Technologies). Illumina mRNAseq libraries were prepared using standard protocols. We sequenced each library from both ends for 76 bp on a lane of a GAII, resulting in 4.8 million paired female reads, 2.6 million paired male reads, and 3.9 million paired mixed-sex larvae reads. The genome was annotated using the MAKER pipeline (Holt and Yandell 2011), which combined SNAP (Korf 2004) and AUGUSTUS (Stanke and Waack 2003) *de novo* gene prediction tools with BLAST homology searches using *D. pseudoobscura* proteins and our mRNAseq experimental evidence preprocessed with Tophat (Trapnell et al. 2009) and Cufflinks (Trapnell et al. 2010). To assess the genome for completeness, we used CEGMA (Parra et al. 2009). We then anchored the scaffolds onto chromosomes based on the *D. pseudoobscura* genome, as in (Zhou and Bachtrog 2012), and scaffolds were stitched together with 500 Ns inserted between scaffold breakpoints.

### Whole Genome Re-Sequencing, Variant Calling and Filtering

For polymorphism analyses, a total of 28 *D. athabasca* iso-female strains were used: 9 Western-Northern, 12 Eastern-A, and 7 Eastern-B. We classified strains into semispecies groups based on a combination of geographic location and courtship song interpulse interval (supplementary tables S1 and S2, Supplementary Material online). Karyotype information was also collected following the method in (Pimpinelli et al. 2010) for each of the 28 lines due to a polymorphic Y-autosome fusion segregating within *D. athabasca* (Miller 1957; Miller and Roy 1964) (supplementary table S2, Supplementary Material online). Genomic DNA was extracted from a single female fly from each of the strains using the same method as above. Single fly Illumina libraries were made and sequenced at Beijing Genome Institute according to the manufacturer’s instructions. We sequenced 90 bp paired-end reads, generating 2 Gb of sequence for each strain.

We aligned the reads from each strain to our reference assembly using Bowtie2 [(Langmead and Salzberg 2012); –very-sensitive], with a high percentage of reads aligning per strain (Mean = 85.3%, SD 1.9%). Mean genomic coverage per strain was 9.19*x* ± 0.38 SD (see supplementary table S2, Supplementary Material online for genome coverage by strain). Variants for each strain were called using the GATK pipeline [version 1.5; (DePristo et al. 2011)]. In brief, PCR duplicates were removed from each strain using Picard (http://picard.sourceforge.net) and strains were merged into a single file. Local realignment was performed on the merged file around indel regions to prevent erroneous variant calls due to alignment error. Variants from all strains were called simultaneously. Due to the lack of validated SNPs in *D. athabasca*, recalibration steps were omitted from the pipeline. Using GATK’s Variant Filtration tool, only those variants that passed our coverage and quality filter were retained (MQ0  > = 4 && ((MQ0/(1.0 * DP)) > 0.1); DP < 5; QUAL < 30.0; QUAL > 30.0 && QUAL < 50.0; QD < 1.5; SB > −10.0). Additionally, we only kept biallelic sites where 5 or more individuals were genotyped per semispecies. Due to a polymorphic Muller C-Y chromosome fusion in *D. athabasca* (Miller and Roy 1964) (supplementary table S2, Supplementary Material online), SNPs on Muller C were omitted from all subsequent analyses. As a method of validation, we performed the variant calling pipeline as described above, including the short-insert reads from the reference strain. We then counted the number of sites in which the reference strain was called as a homozygous variant allele, allowing us to estimate a false-positive rate of 0.009%.

To polarize SNPs, ancestral states for each variant site were assigned by aligning the *D. athabasca* reference genome to the genomes of two closely related species, *D. algonquin* (*D. athabasca − D. algonquin* D_xy_ = 3.9%) and *D. affinis* (*D. athabasca − D. affinis* D_xy_ = 4.3%). Only those variant sites in which both *D. algonquin* and *D. affinis* were aligned and shared the same allele were polarized (68.1%). The *D. algonquin* genome was sequenced from a single Illumina ∼500 bp short insert library. Genomic DNA was extracted from a pool of 10 female flies from a single strain obtained from New Hampshire (NH-2). DNA extraction, library preparation, and Illumina sequencing protocols are identical to those for the *D. athabasca* reference genome. SOAPdenovo (kmer = 29) was used to assemble the reads (28.1 million paired-end, 101 bp reads) into scaffolds, resulting in an assembly with 254,588 scaffolds and total genome size of 165.0 Mb. The scaffold N50 for the *D. algonquin* assembly was 1.8 kb. The *D. affinis* genome was kindly provided by Nicola Palmieri. Outgroup genomes were aligned to the *D. athabasca* reference genome using the LASTZ pipeline (Harris 2007).

### Measurements of Genomic Diversity, Divergence and Population Structure

We calculated standard population genetic statistics for the X (Muller A, AD) and the autosomes (Muller B, E, F). We used PopGenome (Pfeifer et al. 2014) to estimate diversity (π) and absolute divergence (Dxy) in 10 kb non-overlapping windows. To characterize genetic differentiation, we calculated Weir and Cockerhams Fst using VCFtools and the same window size (Danecek et al. 2011). To alleviate any spurious genomic patterns in the data that could arise from anchoring *D. athabasca* scaffolds to the divergent *D. pseudoobscura* genome (17 million years; Beckenbach et al. 1993), we constrained our 10 kb windows to only *D. athabasca* scaffolds from the initial genome assembly prior to anchoring them to *D. pseudoobscura*.

We constructed phylogenetic trees by first partitioning the genome into longer non-overlapping 50 kb windows. We then used RAxML (Stamatakis 2014) to construct maximum likelihood trees with a GTR model and 100 bootstrap replicates. Analyzing the full set of trees was done in R (R Development Core Team 2011) following methods outlined in (Osborne et al. 2016). Briefly, each tree for each region was pruned using *pruneTree* from the *phangorn* package (Schliep 2011), and nodes with < 60% bootstrap support were collapsed. Further, only trees with > 2 well-supported nodes were kept. We then made each tree ultrametric using *chronos* in APE (Paradis et al. 2004) and visualized the full set of trees using *densiTree* from *phangorn* with scaleX = TRUE.

To further assess population structure we used ADMIXTURE (Alexander et al. 2009). To correct for the effects of linkage disequilibrium, we used VCFtools (Danecek et al. 2011) to thin the SNP datasets (above) by extracting SNPs > 1000 bp away from each other. ADMIXTURE analyses were then run with 10-fold cross-validation and *K* values of 1–8. The best *K* was determined as the model with the lowest cross-validation error (Alexander et al. 2009). We also examined clustering of individuals within *D. athabasca* using principal component analysis (PCA). The PCA was implemented on the same set of thinned SNPs as our ADMIXTURE analyses (above) and carried out using the program SMARTPCA (altnormstyle: NO, numoutevec: 10, numoutlieriter: 5, numoutlierevec: 10, outliersigmathresh: 6, qtmode: 0) (Patterson et al. 2006). PCAs were done on covariance of SNPs normalized as described in (Price et al. 2006). After thinning, we used a total of 48,412 X-linked and 57,743 autosomal SNPs for the PCAs.

### Demographic Analyses

To infer demographic parameters in *D. athabasca*, we used the software package ∂a∂i (Gutenkunst et al. 2009). This approach allows for simultaneous demographic inference of up to three populations based on the joint site-frequency spectra (SFS) of the sequences, grouped by semispecies. ∂a∂i uses a Wright–Fisher diffusion approximation method to generate an expected joint SFS under a specified demographic model and compares it to the SFS from the experimental data using a composite likelihood function. We used all autosomal (Muller B, E, F) and X-linked (Muller A, AD) biallelic 4-fold synonymous sites as putative neutral sites for this analysis (95.1 and 49.7 kb). Ancestral states were assigned by polarizing SNPs using alignments to *D. affinis* and *D. algonquin* and sites with missing data were omitted. We tested the fit of our data to an isolation with no migration and an isolation with symmetric migration model, both under a three-population divergence scenario with splitting orders based on the results from clustering analyses (see Results). We used the point estimates from ∂a∂i for the best fitting models with and without migration to generate 100 simulated datasets with the coalescent simulator *ms* (Hudson 2002) and analyzed them with ∂a∂i to obtain standard deviation and confidence interval measurements for demographic parameter estimates. 95% confidence intervals were constructed empirically, as in McCoy et al. (2014). We scaled the maximum likelihood parameter estimates assuming 10 generations per year with the neutral mutation rate estimated from *Drosophila melanogaster* mutation accumulation lines, *μ* = 5.8 × 10^−9^ (Haag-Liautard et al. 2007). A likelihood ratio test was used to compare the fit of the models to the data. Note that neither of these simple models is likely to capture the full history of the *D. athabasca* group. However, examining the goodness-of-fit of our data to these models will increase our understanding of demographic processes within the species group and thus provide an important evolutionary framework for further investigation in this system.

Given our PCA and ∂a∂i results (see Results), we further explored signals of recent introgression between the semispecies using the *f*_3_ statistic in treemix (Pickrell and Pritchard 2012). The *f*_3_ statistic is similar to the D-statistic (or ABBA–BABA test) (Green et al. 2010; Durand et al. 2011) and tests for introgression using a three population tree (Reich et al. 2009). A significantly negative *f*_3_ statistic provides evidence of introgression in the target population from two source populations (Reich et al. 2009). We therefore tested for introgression in each semispecies using all possible trees.

## Results

### Behavioral Classification of Samples into Semispecies Using Courtship Song Differences

We collected population samples from across the *D. athabasca* species range and measured the IPI of High-Repetition-Rate (HRR) song bursts from courtship song recordings. Combining IPI data with geographic range data, we were able to unambiguously assign iso-female lines to specific semispecies groups (see supplementary table S2, Supplementary Material online for IPI averages by line). Average interpulse intervals by semispecies were 11.2 ± 0.8 ms for Western-Northern lines, 29.0 ± 2.6 ms for Eastern-A lines, and 13.4 ± 1.0 ms for Eastern-B lines (fig. 1*a*).

### Reference Genome Assembly and Annotation

Our final draft assembly was 157.2 Mb in size, which is within the range of previously sequenced *Drosophila* species (130–364 Mb; Drosophila 12 Genomes Consortium 2007), and had an N50 of 83.5 kb (table 1). There were a total of 21,028 gaps in the assembly, with a mean gap length of 531.1 bp (SD = 864.7 bp). The total percentage of the genome with informative sequence information was 92.9%. Supplementary table S3, Supplementary Material online shows the size of the ordered and stitched assembly. Our final genome annotation contained 13,378 genes. Similar numbers of protein coding genes have been reported in other *Drosophila* species (13,425–16,874; Attrill et al. 2016). We examined the genome for completeness using CEGMA and found that 98.0% of core eukaryotic genes were present in our reference genome, with 94.8% of them being complete.

**Table 1:** Reference Genome Assembly for *D. athabasca* with Muller Element Assignment Using BLAST.

Muller Element	# Scaffolds	# Genes	Total Size (Mb)
A	418	2,231	26.5
A/D	414	2,407	26.6
B	1,742	2,623	29.5
C	912	2,368	21.6
E	1,285	3,054	33.7
F	20	78	1.2
Unknown	1,860	616	18.1
Total	6,651	13,378	157.2

Note.— Unknown category corresponds to scaffolds labeled “unknown” in the *D. pseudoobscura* assembly.

### Population Resequencing

We re-sequenced individuals from 28 lines distributed widely across the species range (9 Western-Northern, 12 Eastern-A, and 7 Eastern-B), with a mean coverage of 9.19*x* per line (0.38 SD; see supplementary table S2, Supplementary Material online for average depth of genomic coverage for each line). After filtering, our whole genome analysis of *D. athabasca* resulted in a total of 6.6 Mbp of biallelic sites that were variable within *D. athabasca* with at least five genotypes per semispecies. For the analyses requiring polarization, after screening out sites that lacked ancestral state information and any missing data, we were left with a total of 3.2 Mbp of variable sites.

**Fig. 2:**
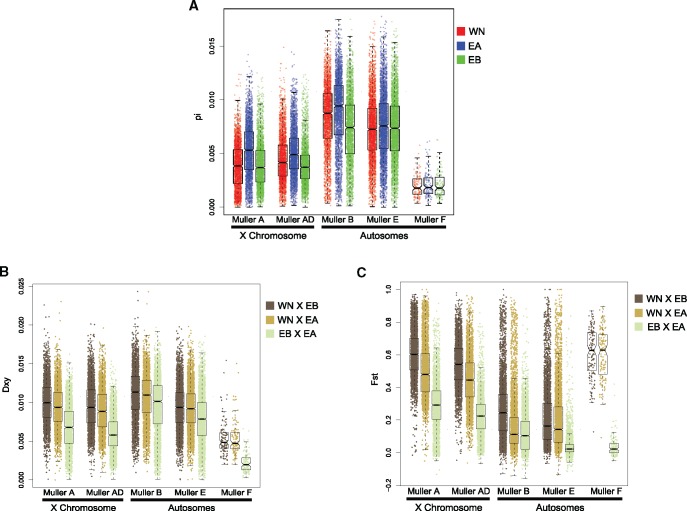
Population genetic estimates for *D. athabasca*. (**a**) Nucleotide diversity within semispecies, and **(b)** Dxy, and **(c)** Fst among semispecies for each Muller element, estimated across the genome in 10 kb windows.

Nucleotide diversity was found to be similar in the three semispecies but lower on the X chromosome (table 2, fig. 2*a*, supplementary tables S4 and S5, Supplementary Material online), which is likely driven by its smaller effective population size. Reduced nucleotide diversity on the X has been observed repeatedly in other Drosophila (Garrigan et al. 2012), including in *D. athabasca* (Ford and Aquadro 1996).

**Table 2:** Estimates of Nucleotide Diversity (π) across the X-Chromosome and Autosomes for Each Semispecies.

Semispecies	X-chromosome	Autosomes	P*
Western-Northern	0.00417	0.00763	<0.0001
Eastern-A	0.00522	0.00804	<0.0001
Eastern-B	0.00398	0.00720	<0.0001

* *P*-value for X vs. autosome comparisons, Mann–Whitney *U*.

### Population structure in *D. athabasca*

**Fig. 3:**
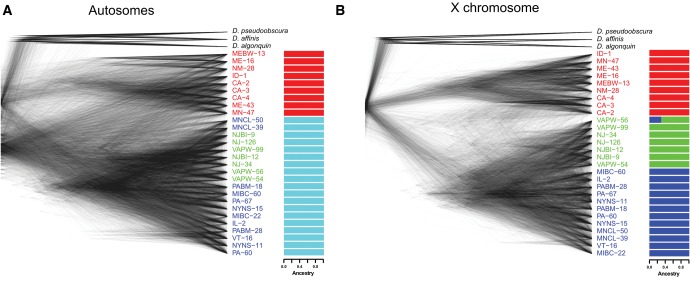
Phylogenetic relationships and population structure among *D. athabasca* semispecies. Maximum likelihood trees for non-overlapping 50 kb windows along the genome and results from ADMIXTURE analysis for the (**a**) autosomes and (**b**) X chromosome. Individuals are color coded by semispecies song type.

Examining inferred phylogenetic trees allows us to identify evolutionary relationships among individuals, independent of predefined classifications. Phylogenetic patterns concordant with behavioral semispecies classifications would provide genetic support for grouping individuals into semispecies despite their young age and the potential for gene flow and/or incomplete lineage sorting. Consistent with the recent formation of these semispecies, autosomal trees were often unresolved and only two groups were identified: WN was found to be somewhat distinct, while EA and EB were indistinguishable (fig. 3*a*). In contrast, phylogenetic relationships inferred from regions of the X chromosome were far better resolved and identified the three behavioral semispecies (fig. 3*b*). To further explore population structure among the semispecies we performed ADMIXTURE analyses. We found groupings consistent with our tree-based analyses: two distinct groups (WN and EA + EB) were identified with autosomal SNPs, while the three distinct behavioral races (WN, EA, and EB) were identified with X-linked SNPs (fig. 3).

**Fig. 4:**
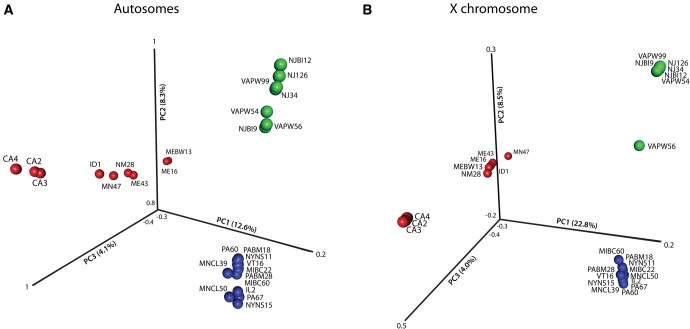
Principal component analysis of X-linked and autosomal SNPs in *D. athabasca*. (**a**) Principal component analysis of autosomal SNPs. First principal component shows a clear separation of WN (red) from both Eastern semispecies but no differentiation between EA (blue) and EB (green). PC2 shows separation of EA and EB, while PC3 suggests geographic structure within WN. (**b**) Principal component analysis of X-linked SNPs shows similar patterns as autosomal SNPs, however PC1, which separates WN from the two Eastern semispecies, explains a much larger amount of the variation.

**Fig. 5:**
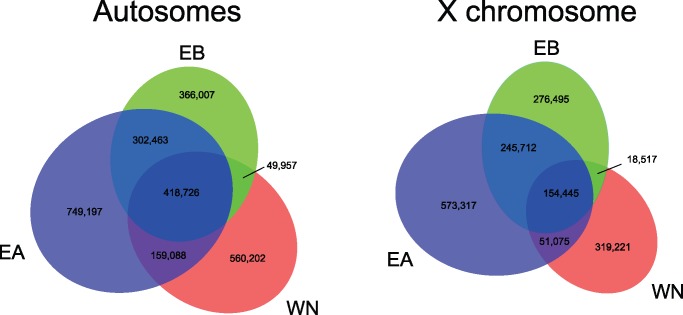
Shared and private X-linked and autosomal SNPs in *D. athabasca*. (**a**) Venn diagram of autosomal SNPs. Most SNP’s are private to each semispecies, followed by SNP’s shared among all three semispecies. (**b**) Venn diagram of X-linked SNPs. Most SNPs are private to each semispecies, followed by SNPs shared between the two Eastern semispecies.

PCA also revealed three distinct clusters corresponding to the three semispecies of *D. athabasca* (PC1 and PC2; fig. 4), both when using SNPs derived from the X chromosome as well as from the autosome. PC3 reveals additional geographic structure in Western-Northern (especially on the autosomes), with the samples from California clustering on one end and Maine clustering on the other (fig. 4*a*); a larger sample size would help to clarify this signal. Consistent with the phylogeny and ADMIXTURE analysis, however, we find that a larger percentage of the variation is explained by X-linked SNPs compared with the autosomal PCA (fig. 4). Genome-wide average estimates of D_xy_ and F_ST_ also strongly point to a closer genetic relationship between Eastern-A and Eastern-B individuals (table 3; fig. 2*b* and *c*, supplementary tables S6 and S7). Note that while F_ST_ is significantly higher for the X relative to autosomes for all three semispecies comparisons, D_xy_ is similar between X-linked and autosomal loci (D_xy_ is in fact slightly lower for the X; table 3; fig. 2*b* and *c*;supplementary tables S6 and S7, Supplementary Material online). Thus, increased population differentiation among semispecies, as measured by F_ST_, is largely driven by reduced levels of polymorphism on the X chromosome relative to autosomes, instead of increased levels of divergence (Charlesworth 1998). Consistent with their recent divergence, a large fraction of SNPs are shared among the three semispecies (25–37% for autosomes, 15–28% for the X). However, the two Eastern semispecies share substantially more SNPs than either does with Western-Northern (fig. 5).

**Table 3:** Estimates of Absolute Divergence (Dxy) and Population Differentiation (F_ST_) for the X-Chromosome and Autosomes.

Statistic	Comparison	X-chromosome	Autosomes	P*
Dxy	WN-EA	0.0092	0.0100	<0.0001
	WN-EB	0.0098	0.0099	<0.0024
	EA-EB	0.0066	0.0087	<0.0001
Fst	WN-EA	0.4201	0.1832	<0.0001
	WN-EB	0.5742	0.1961	<0.0001
	EA-EB	0.2545	0.0915	<0.0001

* *P*-value for X vs. autosome comparisons, Mann–Whitney *U*.

Previous studies have suggested a splitting order for the semispecies of *D. athabasca* where Eastern-A and Eastern-B are more recently diverged sister groups, with Western-Northern having diverged earlier in the genealogical history of the species (Ford et al. 1994; Yoon and Aquadro 1994; Ford and Aquadro 1996). Our dataset also supports this relationship, with both phylogenetic and principal component analyses consistently clustering the Eastern groups together. Additionally, low levels of relative population differentiation and absolute divergence between Eastern semispecies (measured by F_ST_ and D_xy_; table 3; fig. 2*b* and *c*) is indicative of recent shared ancestry and consistent with a (Western-Northern, (Eastern-A, Eastern-B)) splitting model within *D. athabasca*.

### Demographic Analyses

**Fig. 6:**
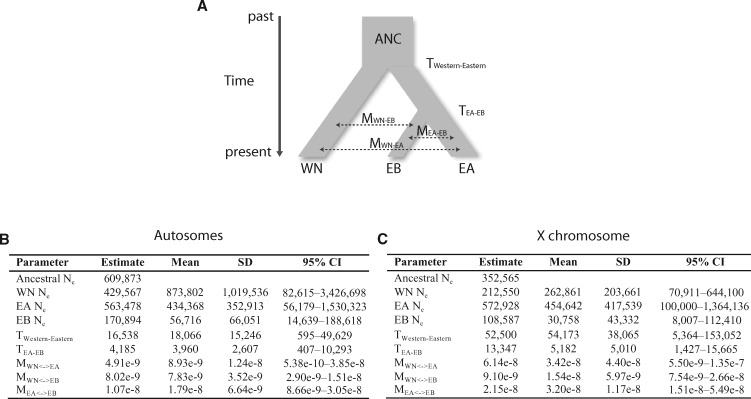
Demographic estimates for the *D. athabasca* complex. (**a**) Divergence model for *D. athabasca* demographic history along with ∂a∂i maximum likelihood estimates of population demographic parameters under an isolation with symmetric migration model inferred from (**b**) autosomal and (**c**) X-linked 4-fold synonymous sites. Mean, standard deviation, and 95% confidence intervals are derived from *ms* simulations. Migration rates are scaled per generation.

We used the software package ∂a∂i (Gutenkunst et al. 2009) for demographic inferences in the *D. athabasca* semispecies complex. Because we were interested in determining whether or not the semispecies of *D. athabasca* diverged with or without gene flow, we tested the fit of our data to an isolation with no migration (allopatric divergence) and an isolation with symmetric migration model, both under a three-population divergence scenario with splitting orders based on the results from clustering analyses (fig. 6*a*). Maximum likelihood estimates of inferred demographic parameters, along with their confidence intervals inferred from simulations are shown in figure 6*b* and *c*. The results from our ∂a∂i analyses suggest that out of the two models we tested, the model that included gene flow (isolation with migration model; fig. 6*a*) fits our data significantly better than the strictly allopatric model for both autosomal and X-linked sites (supplementary fig. S1, Supplementary Material online; Likelihood-ratio-test, Autosomes *X*^2^ = 6.1E + 4, *P* <0.001; X chromosome *X*^2^ = 2.4E + 3, *P* < 0.001). Note however, that inferred migration rates between semispecies are very low (fig. 6*b*). Using our autosomal data, we infer a divergence time of 16,538 years for the Western-Eastern split and 4,185 years for the Eastern-A-Eastern-B split. Inferences using X-linked data resulted in older divergence times, 52,500 years for the Western-Eastern split and 13,347 years for the Eastern-A-Eastern-B split (but note that the confidence intervals for divergence times estimated from X-linked and autosomal data overlap). Estimates of current effective population sizes are consistent with expectations based on current observed ranges, in which Western-Northern and Eastern-A have larger effective population sizes, while estimates for the effective population size of Eastern-B were the smallest (fig. 6*b* and *c*).

Given evidence for low levels of gene flow from our ∂a∂i analyses and a large number of tree topologies that conflicted with the semispecies tree (especially for the autosomes), we sought to further explore the potential for gene flow between semispecies. To disentangle the role of gene flow from that of incomplete lineage sorting, we calculated the *f*_3_ statistic for the X chromosome and autosomes. In all possible three-taxon combinations, we found no compelling evidence of gene flow in these analyses; all *f*_3_ statistics where non-negative (i.e., no introgression) and had very large Z-scores (supplementary table S8, Supplementary Material online). Thus, the lack of phylogenetic resolution appears to be largely driven by incomplete lineage sorting among the recently diverged semispecies and is not due to ongoing gene flow.

## Discussion

### *D. athabasca* Is One of the Youngest Species Complexes

Understanding the genetic basis underlying the process of speciation, and ultimately biodiversity, is a major goal in evolutionary biology. However, despite recent progress identifying genes contributing to postzygotic isolation and thus maintaining species boundaries, little is known about the genetic basis and evolutionary forces that are important driving the initial evolution of reproductive isolation, and thus speciation. To this end, it is necessary to study populations or young species that are in the process of evolving reproductive barriers (Orr 1995; Noor and Feder 2006; Presgraves 2010; Sobel et al. 2010). Previous work in the *D. athabasca* species complex, using both breeding and behavioral assays, as well as investigation of a limited number of molecular markers has suggested that this group may be an ideal model to study the evolutionary forces driving prezygotic isolation (Miller 1958; Miller and Westphal 1967; Miller et al. 1975; Yoon 1991; Ford et al. 1994; Yoon and Aquadro 1994; Ford 1995; Ford and Aquadro 1996; Yukilevich et al. 2016). Until now, however, it remained unclear how representative these previously examined regions were of the entire genome.

We utilized next-generation sequence data to examine population structure and infer the historical demography within the species complex. Overall, we show that both phylogenetic trees (for X-linked loci; fig. 3) as well as principal component analysis (for both X and autosomal loci; fig. 4) support three distinct genetic clusters corresponding to the three behaviorally defined semispecies of *D. athabasca*. Our whole genome data suggest a nested three-population structure within *D. athabasca*, with the Western-Northern semispecies diverging first and the two Eastern semispecies splitting more recently, consistent with previous studies based on a few loci (Ford et al. 1994; Yoon and Aquadro 1994; Ford and Aquadro 1996). Demographic inference using the joint site-frequency spectra confirms a recent split, placing the divergence time for the Western-Northern semispecies at 16,538–52,500 years ago and the Eastern-A/Eastern-B divergence at only 4,185–13,347 years (fig. 6). Previous estimates by Ford and Aquadro (1996) lie within the standard deviation of our estimates, leaving their proposed model of post-glacial species expansion plausible. The *D. athabasca* species complex is thus one of the youngest systems studied at the genome-wide level to date that has evolved prezygotic isolation, and this study provides an important framework for future evolutionary analyses in this species group.

### Demographic Signatures Are Complex within *D. athabasca*

Although our demographic analysis estimates low levels of migration between the semispecies and our analyses of population structure indicate mixed ancestry on the X-chromosome in one of our samples (VAPW-56; fig. 3*b*), we find little evidence for gene flow using the *f*_3_ statistic. Previous studies exploring mtDNA haplotype sharing in *D. athabasca* also found no evidence for gene flow between semispecies (Yoon and Aquadro 1994). In order to disentangle these somewhat conflicting signals, further research is needed with additional individuals to test for ancestral or ongoing gene flow among semispecies of *D. athabasca*.

Previous studies have suggested the possibility that the formation of the Eastern-B semispecies may have been the result of a founder event (Ford and Aquadro 1996). Our analyses, however, yield inconsistent signals. We estimate a reduced effective population size for Eastern-B using the allele-frequency spectrum, consistent with a potential founder event. However, levels of nucleotide diversity in Eastern-B only show a slight reduction across the genome compared with the other semispecies. The small sample size (*n* = 7) for Eastern-B could potentially downwardly bias our estimates of effective population size (Keinan and Clark 2012). Again, sampling of additional individuals and more complex demographic models are needed to better understand the population history of *D. athabasca.*

### Differences in Population Structure on the X Versus Autosomes within *D. athabasca*

We find varying patterns of differentiation on the X-chromosome vs. autosomes, with the X-chromosome showing higher levels of phylogenetic resolution and stronger genetic clustering of semispecies. We also show that levels of population differentiation (F_ST_) between semispecies are elevated on the X-chromosome, confirming previous work (Ford and Aquadro 1996), and inferred population split times among semispecies are more distant for X-linked loci. Incomplete lineage sorting results in unresolved species trees (Degnan and Salter 2005; Degnan and Rosenberg 2009), and the probability that incomplete lineage sorting affects a locus depends on its effective population size (Pamilo and Nei 1988). We thus expect loci on the X chromosome, which has a reduced effective population size (and less diversity) relative to autosomes to more accurately reflect the true species tree.

Inversions may play a special role during speciation and local adaptation by shielding adaptive differences from recombination and may thus lead to elevated divergence among populations (Kirkpatrick 2010). Studies have mapped loci known to be involved in reproductive isolation and local adaptation to inverted regions in multiple species groups that have experienced recent introgression, including *Drosophila* (Noor et al. 2001; Khadem et al. 2011), monkeyflowers (Lowry and Willis 2010; Fishman et al. 2013), sunflowers (Kim and Rieseberg 1999), sticklebacks (Jones et al. 2012), and butterflies (Joron et al. 2011). Interestingly, previous studies investigating variation in salivary gland chromosomes have found a number of polymorphic and fixed inversions within *D. athabasca* (Novitski 1946; Miller and Sanger 1968; Miller and Voelker 1968, 1969a,b, 1972), and a total of over 70 inversions across all semispecies of *D. athabasca* have been inferred using cytological methods (Johnson 1985). Specifically, the X chromosome was reported to harbor seven fixed inversions between Western and Eastern semispecies, and an additional three fixed inversions separate Eastern-A and Eastern-B semispecies (Yoon and Aquadro 1994). However, fixed inversions are not unique to the X chromosomes, and several of the autosomes were found to harbor a similar (or larger) number of fixed inversions among semispecies (Johnson 1985). Investigating whether inversions along the X chromosome contribute to overall patterns of increased X-linked divergence in *D. athabasca* requires precise mapping of the inversions. However, we were unable to confidently identify the previous cytologically reported inversions in *D. athabasca* using our fragmented genome assembly combined with our short-read data and current methods. Future work using longer read technologies and improved assemblies should help to clarify the role inversions might have played in the *D. athabasca* divergence.

Elevated divergence on the X-chromosome could also suggest that it plays an important role in population differentiation within this species complex. Increased divergence along the X chromosome in other systems has been attributed to the large X-effect, which is classically thought of the X chromosome being a hotspot for hybrid male sterility factors (Coyne and Orr 1989; Coyne 1992; Presgraves 2008). Species experiencing gene flow via hybridization may therefore accumulate divergence more rapidly on the X, since introgression on the X chromosome is less likely than on autosomes because of its higher density of hybrid male sterility factors (Moyle et al. 2010). However, this cannot be the case in *D. athabasca* since hybrids between semispecies are fertile, suggesting the X chromosome may be of broader importance during speciation, beyond hybrid male sterility. Specifically, the presence of “speciation genes” on the X chromosome could contribute to increased divergence among semispecies. As mentioned, *D. athabasca* semispecies show a high degree of sexual isolation but produce fertile offspring with no evidence of hybrid breakdown (Miller 1958; Miller and Westphal 1967; Miller et al. 1975; Yoon 1991; Ford et al. 1994; Yoon and Aquadro 1994; Ford and Aquadro 1996). Male courtship song, and particularly the IPI phenotype differs among *D. athabasca* semispecies, and analysis of backcross hybrids among semispecies showed patterns of segregation of IPI consistent with a major effect on the X chromosome (Yoon 1991; Yukilevich et al. 2016). Thus, the presence of behavioral isolation genes (i.e., male courtship song genes and possibly female preference genes) on the X, perhaps associated with fixed inversions among semispecies could contribute to elevated divergence on the X relative to autosomes. Again, more contiguous genome assemblies and sampling of more individuals together with mapping studies should help to reveal the nature and location of behavioral isolation genes in *D. athabasca*.

## Conclusions and Future Prospects for *D. athabasca*

*D. athabasca* is a compelling group in which to study incipient speciation. Semispecies share regions of sympatry, exhibit prezygotic isolation, and have very recent divergence times. Previously, speciation studies were mostly limited to classic model organisms for which genomic resources have been well developed, and their closely related sister species. However, next-generation sequencing technologies have opened up the possibility of expanding and developing additional, more pertinent model systems for the study of speciation. The behaviorally distinctive semispecies have long made *D. athabasca* an attractive model for descriptive studies involving prezygotic isolation (Miller 1958; Miller and Westphal 1967; Yoon 1991) and population differentiation (Johnson 1985). However, until now the lack of genomic resources have limited evolutionary investigations in this species group. Our broad genomic survey of the patterns of diversity and population structure within *D. athabasca* provide a first step towards developing important genomic resources and a historical framework necessary for future evolutionary analyses in *D. athabasca*.

## Data Access

The genome assembly is available at the National Center for Biotechnology Information under BioProject ID PRJNA274695. All the DNA/RNA-seq reads generated in this study are deposited at NCBI Short Reads Archive (http://www.ncbi.nlm.nih.gov/sra) under the accession number PRJNA274411.

## Supplementary Material

Supplementary data are available at *Molecular Biology and Evolution* online.

## Supplementary Material

Supplementary Data
