# Supplementary material for: Patterns of Genome-Wide Diversity and Population Structure in the Drosophila athabasca Species Complex
Source: Mol Biol Evol. 2017 Apr 14;34(8):1912–23. doi: 10.1093/molbev/msx134 (PMC5850846; doi:10.1093/molbev/msx134)
Supplement: Supplementary Data [file msx134_supp.docx]

**Supplementary Tables**

**Supplementary Table 1.** Summary of collections and number of *D. athabasca* lines collected at each location.

**Supplementary Table 2.** *D. athabasca* lines used in whole genome resequencing, along with semispecies assignment, interpulse interval, karyotype information, and average depth of genomic coverage.

* Only a single replicate was obtained for these lines

**Supplementary Table 3.** Size of stitched reference genome assembly for *D. athabasca* using *D. pseudoobscura* homology to anchor scaffolds into chromosomes. Stitched genome has been deposited at NCBI under BioProject ID PRJNA274695.

| **Muller Element** | **# Scaffolds** | **Stitched Size (Mb)** |
| --- | --- | --- |
| A | 221 | 20.9 |
| A/D | 290 | 30.4 |
| B | 766 | 25.6 |
| C | 478 | 20.3 |
| E | 636 | 31.8 |
| F | 29 | 1.4 |
| Total | 2420 | 130.4 |

**Supplementary Table 4.** Estimates of nucleotide diversity (π) for synonymous sites (4-fold degenerate) and amino acid sites across the X-chromosome and autosomes for each semispecies.

| **Semispecies** | **Synonymous sites** | |  | **Replacement sites** | |
| --- | --- | --- | --- | --- | --- |
|  | **X-chromosome** | **Autosomes** |  | **X-chromosome** | **Autosomes** |
| Western-Northern | 0.00504 | 0.01332 |  | 0.00095 | 0.00197 |
| Eastern-A | 0.00633 | 0.01391 |  | 0.00113 | 0.00198 |
| Eastern-B | 0.00419 | 0.01174 |  | 0.00093 | 0.00178 |

**Supplementary Table 5.** Nucleotide diversity by Muller element and semispecies.

| **population** | **Muller element** | **pi mean** | **pi median** | **sd** |
| --- | --- | --- | --- | --- |
| WN | Muller A | 0.0038 | 0.0038 | 0.0020 |
| EA | Muller A | 0.0053 | 0.0053 | 0.0024 |
| EB | Muller A | 0.0040 | 0.0037 | 0.0021 |
| WN | Muller AD | 0.0045 | 0.0042 | 0.0022 |
| EA | Muller AD | 0.0051 | 0.0049 | 0.0022 |
| EB | Muller AD | 0.0040 | 0.0037 | 0.0019 |
| WN | Muller B | 0.0083 | 0.0087 | 0.0032 |
| EA | Muller B | 0.0089 | 0.0094 | 0.0034 |
| EB | Muller B | 0.0073 | 0.0074 | 0.0032 |
| WN | Muller E | 0.0073 | 0.0073 | 0.0029 |
| EA | Muller E | 0.0076 | 0.0076 | 0.0031 |
| EB | Muller E | 0.0073 | 0.0074 | 0.0030 |
| WN | Muller F | 0.0020 | 0.0018 | 0.0010 |
| EA | Muller F | 0.0021 | 0.0018 | 0.0012 |
| EB | Muller F | 0.0021 | 0.0018 | 0.0012 |

**Supplementary Table 6.** Dxy by Muller element.

| **comparison** | **Muller element** | **dxy mean** | **dxy median** | **sd** |
| --- | --- | --- | --- | --- |
| EB x WN | Muller A | 0.0101 | 0.0100 | 0.0028 |
| EA x WN | Muller A | 0.0095 | 0.0094 | 0.0027 |
| EA x EB | Muller A | 0.0068 | 0.0068 | 0.0028 |
| EB x WN | Muller AD | 0.0095 | 0.0094 | 0.0029 |
| EA x WN | Muller AD | 0.0090 | 0.0088 | 0.0028 |
| EA x EB | Muller AD | 0.0061 | 0.0058 | 0.0024 |
| EB x WN | Muller B | 0.0111 | 0.0113 | 0.0033 |
| EA x WN | Muller B | 0.0107 | 0.0110 | 0.0031 |
| EA x EB | Muller B | 0.0096 | 0.0101 | 0.0036 |
| EB x WN | Muller E | 0.0094 | 0.0093 | 0.0028 |
| EA x WN | Muller E | 0.0093 | 0.0092 | 0.0028 |
| EA x EB | Muller E | 0.0079 | 0.0079 | 0.0032 |
| EB x WN | Muller F | 0.0054 | 0.0048 | 0.0023 |
| EA x WN | Muller F | 0.0054 | 0.0047 | 0.0022 |
| EA x EB | Muller F | 0.0022 | 0.0020 | 0.0012 |

**Supplementary Table 7.** Fst by Muller element.

| **comparison** | **Muller element** | **Fst mean** | **Fst median** | **sd** |
| --- | --- | --- | --- | --- |
| EB x WN | Muller A | 0.607 | 0.603 | 0.147 |
| EA x WN | Muller A | 0.503 | 0.479 | 0.173 |
| EA x EB | Muller A | 0.294 | 0.292 | 0.154 |
| EB x WN | Muller AD | 0.541 | 0.542 | 0.143 |
| EA x WN | Muller AD | 0.449 | 0.445 | 0.155 |
| EA x EB | Muller AD | 0.228 | 0.224 | 0.126 |
| EB x WN | Muller B | 0.270 | 0.244 | 0.188 |
| EA x WN | Muller B | 0.175 | 0.112 | 0.183 |
| EA x EB | Muller B | 0.124 | 0.104 | 0.121 |
| EB x WN | Muller E | 0.219 | 0.163 | 0.187 |
| EA x WN | Muller E | 0.203 | 0.142 | 0.188 |
| EA x EB | Muller E | 0.037 | 0.022 | 0.067 |
| EB x WN | Muller F | 0.610 | 0.627 | 0.150 |
| EA x WN | Muller F | 0.604 | 0.628 | 0.153 |
| EA x EB | Muller F | 0.038 | 0.022 | 0.066 |

**Supplementary Table 8.** Results from three population tests (*f*_3_) estimating admixture between semispecies.

| **Chromosome** | | **Target** | **Source 1** | **Source 2** | ***f*_3_** | **Standard error** | **Z-score** |
| --- | --- | --- | --- | --- | --- | --- | --- |
| Autosomes | | EA | EB | WN | 0.001 | 0.0001 | 27.18 |
|  |  | EB | EA | WN | 0.011 | 0.0002 | 48.80 |
|  |  | WN | EB | EA | 0.030 | 0.0006 | 51.78 |
| X chromosome | | EA | EB | WN | 0.007 | 0.0002 | 35.78 |
|  |  | EB | EA | WN | 0.034 | 0.0004 | 90.49 |
|  |  | WN | EB | EA | 0.093 | 0.0013 | 70.95 |

**Supplementary Figures**

**Supplementary Figure 1.** ∂a∂i plots comparing the fit of the observed joint site frequency spectra for the autosomes and X chromosomes under **(a,c)** isolation with no migration and **(b,d)** isolation with symmetric migration models. Top row = observed data; second row = expected under the model; bottom row = residuals.
